# Outcome and quality of life after surgically treated ankle fractures in patients 65 years or older

**DOI:** 10.1186/1471-2474-8-127

**Published:** 2007-12-20

**Authors:** Gertrud Nilsson, Kjell Jonsson, Charlotte Ekdahl, Magnus Eneroth

**Affiliations:** 1 Department of Health Sciences, Division of Physical Therapy, Lund University, Lund, Sweden; 2 Primary Healthcare, Research Department, Skane County Council, Malmö, Sweden; 3 Department of Radiology, Lund University Hospital, Lund, Sweden; 4 Department of Orthopaedics, Lund University Hospital, Lund, Sweden

## Abstract

**Background:**

Despite high incidence of ankle fractures in the elderly, studies evaluating outcome and impact of quality of life in this age group specifically are sparse. The aim of this study was to evaluate outcome and quality of life 6 and 12 months after injury in patients 65 years or older who had been operated on due to an ankle fracture.

**Methods:**

Sixty patients 65 years or older were invited to participate in the study. 6 and 12 months after the injury a questionnaire including inquiry to participate, the Olerud-Molander Ankle Score (OMAS), Short-Form 36 (SF-36), Linear Analogue Scale (LAS), Self-rated Ankle Function and some supplementary questions was sent home to the patients. The supplementary questions concerned subjective experience of ankle instability, sporting and physical activity level before injury and recaptured activity level at follow-ups, need of walking aid before injury, state of living before injury and at follow-ups and co-morbidities. After the 12-month follow-up the patients were also called for a radiological examination.

**Results:**

Fifty patients (83%) answered the questionnaire at 6-month and 46 (77%) at the 12-month follow-up. Although, 45 (90%) fractures were low-energy trauma 44 (88%) were bi- or trimalleolar and post-operative reduction results were complete in 23 (46%) ankles. The median OMAS improved from 60 (Interquartile range (IQR) 36) at 6-month to 70 (IQR 35) at 12-month (p = 0.002), but at 12-month still sixty percent or more of the patients reported pain, swelling, problems when stair-climbing and reduced activities of daily life. Twenty (40%) rated their ankle function as 'good' or 'very good' at 6-month and 30 (60%) at 12-month. Forty-one (82%) were physically active before injury but still one year after only 18/41 had returned to their pre-injury physical activity level. According to SF-36 four dimensions differed from the age- and gender matched normative data of the Swedish population, 'physical function', 'role physical' and 'role emotional' were below norms at 6-month for women (p = 0.010, p = 0.024 and 0.031) and 'general health' was above norms at 12-month for men (p = 0.044).

**Conclusion:**

One year after surgically treated ankle fractures a majority of patients continue to have symptoms and reported functional limitations. However, SF-36 scores indicate that only females had functional status below the age- and gender matched normative data of the Swedish population.

## Background

Over the recent decades the incidence of ankle fractures has increased especially among the elderly [1,2]. In Finland the total annual number of ankle fractures due to minimal trauma increased in persons over 60 years of age from 369 (57/100 000) in 1970 to 1545 (159/100 000) in 2000. If this trend continues there will be three times more low-trauma ankle fractures in 2030 [2].

Some studies evaluating subjective recovery have reported only slight disability after surgically treated ankle fractures [3-6]. Although later reports have shown poorer results than has been previously described [7-9]. Day *et al.* showed that 36% of the studied patients had fair or poor outcome 10 years after injury [8] and Lash *et al.* concluded that patients can be expected to experience functional difficulties two years post-treatment [7]. We found in an earlier study of patients aged 18–64 years that pain, stiffness and swelling were reported from more than half of the patients and 40% had problem when using stairs both one and three years after injury even though only 18% had a remaining slight fracture displacement or discrete signs of osteoarthritis [9]. However, all these studies have included all ages, also younger persons and except for in the study by Nilsson *et al.*[9] not divided the study results per age group, which means there is lack of information and outcome after ankle fractures explicit in the elderly.

Age has been reported as a predictor of outcome. An age of more than forty years was found to give lower scored functional recovery as measured by the Olerud-Molander Ankle Score (OMAS) in patients 18–64 years of age [9]. Recently similar results were also found in patients 16–89 years of age by Egol *et al.* using the Short Musculoskeletal Function Assessment (SMFA) [10]. Fink *et al.* studied number of 'bed days' and 'limited activity days' during a mean follow up period of 3.8 years after fractures in postmenopausal women. After an ankle fracture 57% reported one or more 'bed days' (second place after hip fracture) and 90% reported 'limited activity days' (fourth place after hip, vertebral and humerus fractures) [11]. Ho *et al.* studied 18 postmenopausal women one year after injury and found generally satisfactory outcome using the Functional Independence Measure (FIM) [12]. In that study neither subjectively scored ankle function or quality of life was evaluated [12].

As the population of the aged continues to grow in number and the ankle fractures can be expected to increase in the elderly it is important to evaluate outcome in this age group specifically. The aim of this study was to evaluate outcome and quality of life 6 and 12 months after injury in patients 65 years or older who had been operated on due to an ankle fracture.

## Methods

### Subjects

During January 2003 – April 2005, 65 consecutive subjects (44 women and 21 men) 65 years or older were operated on due to an ankle fracture at the Department of Orthopaedics, University Hospital, Lund, Sweden. At the first follow-up 6 months later three patients had died and two were unable to participate due to dementia. Thus 60 patients were invited and 50 (83%), 16 men and 34 women, accepted to participate and answered the questionnaire at 6-month follow-up. At 12-month 46 (77%) subjects answered and radiographic examination was performed in 42 (70%).

### Design

In this case-series a descriptive design was used. Six months after the injury a letter, including information about the study, inquiry to participate and a questionnaire, was sent home to the patients. When accepted to participate written informed consent had to be returned in a prepaid envelope together with the completed questionnaire. One reminder was sent after two weeks to those who did not answer. A new questionnaire including the same questionnaire material was sent home after another 6 months. After the 12-month follow-up the patients were also called for a radiographic examination. The Research Ethics Committee at the Lund University approved the study (LU 513-03).

### Outcomes

#### Fracture characteristics and treatment

Information like injured side, type of trauma, type of fracture, type of surgery, number of days spent in hospital, plaster time, weight bearing during plaster time, co-morbidities and complications were registered in standardised protocols.

#### Olerud-Molander Ankle Score

The Olerud-Molander Ankle Score (OMAS) is a self-administered patient questionnaire. The scale is a functional rating scale from 0 (totally impaired) to 100 (completely unimpaired) and is based on nine different items: pain, stiffness, swelling, stair climbing, running, jumping, squatting, supports and activities of daily living. OMAS has been frequently used to evaluate subjectively scored function after ankle fracture [3,6,13,14]. The score is validated against (a) linear analogue scale (LAS) measuring subjective recovery, (b) range of motion in loaded dorsal extension, (c) presence of osteoarthritis and (d) presence of dislocations on radiographs, and it has been found to correlate well with these four parameters [13]. No floor or ceiling effects have been reported [15].

#### Linear Analogue Scale

The patients had to mark their subjective ankle function on a 15 cm long linear analogue scale (LAS) with the ends marked 'perfectly normal function' and 'worst possible function'. The distance between the end of the scale and the mark was measured and was registered as a percentage of 'perfectly normal function', which was graded as 100% [13]. LAS has been validated against OMAS in patients with surgically treated ankle fractures and was found to correlate well with this ankle score [13].

#### Self-rated ankle function

The patients were asked to evaluate their present ankle function as 'very good' (score = 1), 'good' (score = 2), 'fair' (score = 3), 'poor' (score = 4) and 'very poor' (score = 5).

#### Short-Form 36

The Short-Form 36 (SF-36) is a self-administered generic questionnaire designed to evaluate health-related quality of life. The instrument measures eight health domains using eight scales assessing physical function (PF), role limitation due to physical problems (RP), bodily pain (BP), general health (GH), vitality (VT), social function (SF), role limitation due to emotional problems (RE), and mental health (MH). The subscales range from 0–100 and low score implies poor health status, high score implies good health status [16]. The SF-36 has been validated for use in Sweden and normative data on healthy people have been reported [17]. No studies evaluating reliability and validity of SF-36 for use in people with ankle fractures have been found. However, SF-36 is widely accepted as a generic outcome measure and has been employed in some studies concerning patients with ankle fractures [6,18-21]. In the present study, norms of the Swedish population for males and women 65 years or older were compared to patients' results [22].

#### Supplementary questions

The questionnaire was supplemented with additional questions concerning subjective experience of ankle instability. The answering alternatives were: none; 1–2 episodes/year (during exercise); 1–2 episodes/month (during exercise); when walking on uneven ground; when walking on even ground; constant (severe) using ankle support [23]. Sporting or physical activity level before injury and recaptured physical activity level at follow-ups had to be filled in. The information given was then transformed into the Physical Activity Scale by Grimby by the investigator (GN) [24]. The other questions concerned the need of and if so type of walking aid before the injury, state of living before injury and at follow-ups, co-morbidities and if having visited a physiotherapist after plaster removal.

#### Radiographic examination

Radiographic examination including ankle joint congruency, fracture healing, fracture reduction and presence of osteoarthritis (loss of joint space less than 50%; loss of joint space more than 50% but no bone-to-bone contact; bone-to-bone contact) was performed pre-surgery, post-surgery and median 16 (range 12–32 (IQR 8.3)) months post-surgery. All radiographs were examined by the same radiologist (KJ). Results from radiographic examination were registered in standardised protocols.

### Statistics

Statistical analyses were performed using SPSS software, version 11.5. Continuous variables were checked regarding assumptions underlying parametric and nonparametric statistics, and were described and analysed accordingly. To analyse differences between 6-month and 12-month follow-up regarding OMAS, LAS and self-rated ankle function the Wilcoxon's Signed Rank test was used. To analyse differences between men and women regarding age, OMAS, LAS and self-rated ankle function the Mann-Whitney *U*-test was employed. When comparing the results from SF-36 to the norms of the age- and gender matched Swedish population the Independent-Samples *t*-test was used. In analysing the differences between men and women regarding type of fracture, fracture reduction results and presence of osteoarthritis the Chi-square test was used. Spearman's coefficient of correlation was used for analysis between OMAS and LAS. An alpha level of < 0.05 was regarded as significant.

## Results

### Patient characteristics

The age of women was median 72 years (range 65–89) (IQR 14) and of men 71 (range 66–80) (IQR 6). There was no difference regarding age between men and women (p = 0.417). Thirty-nine (78%) patients had one or more co-morbidities and of those 21 had impairments and disabilities in the musculoskeletal system, of those 10 were prior surgeries due to: hip-fracture (n = 1), hip-arthritis (n = 5), knee-arthritis (n = 3) and foot-fracture and hallux valgus (n = 1). Four patients had diabetes.

### Fracture characteristics and treatment

Forty-five (90%) of the fractures were low-energy trauma; 31 had stumbled or slipped on the ground or in a slope and 9 had stumbled in a staircase, five were minor bicycle accidents. Three fractures had occurred when falling from height and were thereby classified as high-energy trauma. In two cases the information was missing. The left ankle was injured in 28 subjects and the fractures were classified as 39 SE IV (supination-eversion grade IV), 2 SE III, 2 SE II, 1 SA II (supination-adduction), 1 SA I, 2 PE IV (pronation-eversion), 2 PE III and 1 PA II (pronation-abduction) [25] with no differences between men and women (p = 0.442). Twenty-six ankles were treated with internal fixation as described by Wiberg-Cedell [26], four patients with the method described by the AO group [27] and 14 patients with a combination of these two methods. Five patients were operated with percutanous fixation due to open fractures. One patient was initially planned for surgical treatment of the fracture but due to other diseases non-surgical intervention had to be chosen. This patient was included in the study. Superficial wound infection occurred in one patient, two had a deep infection and one had deep infection and also local skin necrosis (Table 1).

**Table 1:** Fracture characteristics and treatment in subjects 65 years or older with surgically treated ankle fracture

**Variables**	**Subjects n = 50**
**Gender**	
- men	34
- women	16
**Injured side**	
- left	28
- right	22
**Type of trauma**	
- low energy	45
- high energy	3
- missing	2
Type of fracture*	
- SE IV	39
- SE III	2
- SE II	2
- SA II	1
- SA I	1
- PE IV	2
- PE III	2
- PA II	1
**Operation technique**	
- Wiberg-Cedell	26
- AO	4
- mixed technique	14
- percutan fixation	5
- non-sugical	1
**Complications**	
- no complications	48
- superficial infection	1
- deep infection and skin necrosis	1

*SE = supination-eversion; SA = supination-adduction PE = pronation-eversion; PA = pronation-abduction

All patients were given a below knee plaster cast and the median time in plaster was 43 (range 37–125 (IQR 6)) days. During the first two weeks after surgery 43 (86%) patients were prescribed non-weight bearing and during the next four weeks 29 (58%) had the same prescription. After 6 weeks there were still 12 (24%) persons who were ordered not full weight bearing on their fractured leg. Eight patients had another surgical procedure, all due to symptoms related to the osteosynthetic material.

Median hospital stay was five (range 2–62 (IQR 3.5)) days. All but one had returned to the same state of living as before injury at the 6-month follow-up, although 17 (34%) patients needed care for between one and ten weeks in a nursing home before being able to return back to their ordinary living. After plaster removal 19 patients were reviewed between one and five times at the orthopaedic clinic, in 12 cases the visits were initiated by the doctor as check-ups and in 7 cases initiated by the patients mostly due to pain. Twenty-eight patients (56%) had visited a physiotherapist.

### Olerud-Molander Ankle Score

The first follow-up occurred 6.8 (SD 0.69) months after surgery and the median OMAS at that time was 60 (IQR 36.25). The last follow-up occurred 12.7 (SD 0.71) months after surgery and the corresponding figures at that time was 70 (IQR 35). In the total sample and in women there was a significant improvement between the two follow-ups (p = 0.002) and (p = 0.005) (Table 2). There was no difference between men and women neither at 6-month (p = 0.133) nor at 12 month (p = 0.188). Some sort of pain was reported from 35 (70%) subjects at 6-month and from 27 (59%) at 12-month and mostly when walking on uneven surface. More than half of the subjects experienced stiffness. About 75% reported swelling and problems when stair-climbing at 6-month and still at 12-month more than half of the patients had reduced activities of daily living compared to pre-injury (Table 3).

**Table 2:** Results from Olerud-Molander Ankle Score (OMAS), Linear Analogue Scale (LAS) and Self-rated ankle function 6 and 12-months after surgically treated ankle fracture

	**6-month follow-up **Median (IQR)	**12-month follow-up **Median (IQR)	**p-value**
**OMAS (0–100)**			
- women (n = 32)	55.0 (36.0)	65.0 (28.75)	**0.005**
- men (n = 14)	67.5 (36.25)	80.0 (28.75)	0.152
- total (n = 46)	60.0 (36.25)	70.0 (35.0)	**0.002**
**LAS (0–100)**			
- women (n = 29)	71.0 (32.5)	85.0 (36.0)	**0.011**
- men (n = 14)	75.5 (18.5)	80.5 (37.5)	0.387
- total (n = 43)	74.0 (28.0)	83.0 (33.0)	**0.010**
Self-rated ankle function (1–5)*			
- women (n = 32)	3.0 (1.0)	2.0 (1.75)	0.060
- men (n = 14)	2.5 (1.0)	2.5 (2.0)	0.480
- total (n = 46)	3.0 (1.0)	2.0 (2.0)	**0.049**

IQR = Interquartile range; * 1 = very good; 2 = good; 3 = fair; 4 = poor; 5 = very poor

**Table 3:** Frequencies of patients and scoring weights from the nine domains of Olerud-Molander Ankle Score (OMAS) 6 and 12 months after surgically treated ankle fracture

**Items**	**OMAS 6-month **n = 50	**OMAS 12-month **n = 46	**Scoring weights**
**Pain**			
- None	15	19	25
- While walking on uneven surface	26	21	20
- While walking on even surface outdoors	2	2	10
- While walking indoors	2	3	5
- Constant and severe	5	1	0
**Stiffness**			
- None	21	23	10
- Stiffness	29	23	0
**Swelling**			
- None	12	18	10
- Only evenings	30	23	5
- Constant	8	5	0
**Stair-climbing**			
- No problems	13	16	10
- Impaired	33	27	5
- Impossible	4	3	0
**Running**			
- Possible	12	13	5
- Impossible	38	33	0
**Jumping**			
- Possible	12	11	5
- Impossible	38	34	0
**Squatting**			
- No problems	20	18	5
- Impossible	30	28	0
**Support**			
- None	28	31	10
- Taping, wrapping	7	4	5
- Stick or crutch	7	4	0
- Walking frame	7	7	0
- Wheel-chair	1		0
**Activities of daily life**			
- Same as before injury	21	18	20
- Loss of tempo	12	14	15
- Change to simpler activity	14	12	10
- Severely impaired activity	3	2	0

### Linear Analogue Scale

The median Linear Analogue Scale (LAS) at 6-month was 74 (IQR 28) and at 12 month 83 (IQR 33). There was a significant improvement between the two follow-ups in the total sample p = 0.010) and in women (p = 0.011) (Table 2). There was no difference between men and women neither at 6-month (p = 0.686) nor at 12 month (p = 0.562). A correlation of r_s _= 0.68 (p < 0.001) was found between OMAS and LAS at 6-month and 0.72 (p < 0.001) at 12-month.

### Self-rated ankle function

**Figure 1:**
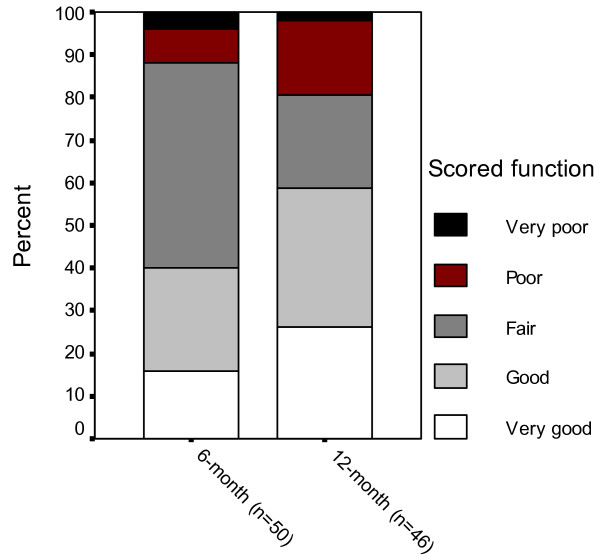
**Self-rated ankle function**. Self-rated ankle function 6 and 12 months after surgically treated ankle fracture in patients 65 years or older.

The median value for self-rated ankle function had increased from 3 (fair) to 2 (good) between the 6- and 12-month follow-up in the total sample (p = 0.049) (Table 2). Eight patients scored 'very good' function at 6-month follow-up, 12 scored 'good', 24 scored 'fair', 4 scored 'poor' and 2 scored 'very poor'. The corresponding figures at 12-month were 12 'very good', 15 'good', 10 'fair', 8 'poor' and 1 'very poor' (Figure 1).

### Short-form SF-36

The women deviated from the age-and gender matched normative data of the general population regarding 'physical function' (p = 0.010), 'role physical' (p = 0.024) and 'role emotional' (p = 0.031) at 6-month follow-up showing impaired results. The differences were eliminated at 12-month (Table 4). No deviations were found in men at 6-month follow-up but at 12-month the men deviated from the normative data regarding 'general health' (p = 0.044) showing improved results in the patient group (Table 5).

**Table 4:** SF-36 in women over 65 years of age 6 and 12 months after surgically treated ankle fracture and the age- and gender matched norms of the Swedish population

**SF-36 sub-scales**	**6-month follow-up **(n = 31) Mean (SD)	**12-month follow-up **(n = 31) Mean (SD)	**Norms of the Swedish population **Mean (95% CI)	p-values* 6-month follow-up	**p-values¤ 12-month follow-up**
**Physical functioning**	52.7 (27.6)	60.3 (27.4)	66.3 (64.0–68.5)	**0.010**	0.278
**Role physical**	42.7 (39.9)	51.6 (43.7)	59.8 (56.2–63.5)	**0.024**	0.217
**Bodily pain**	64.6 (22.5)	73.2 (25.4)	65.7 (63.3–68.0)	0.796	0.134
**General health**	65.2 (20.0)	67.4 (25.4)	63.0 (60.9–65.1)	0.559	0.519
**Vitality**	61.6 (20.6)	61.4 (20.9)	64.2 (61.9–66.4)	0.490	0.487
**Social functioning**	79.0 (26.9)	79.8 (25.8)	82.9 (80.9–84.8)	0.429	0.389
**Role emotional**	53.8 (46.9)	68.8 (44.71)	72.8 (69.4–76.2)	**0.031**	0.452
**Mental health**	73.5 (22.4)	74.3 (22.0)	77.3 (75.4–79.2)	0.358	0.457

* p-values comparing patients results to normative data at 6-month follow-up;

¤ p-values comparing patients results to normative data at 12-month follow-up

**Table 5:** SF-36 in men over 65 years of age 6 and 12 months after surgically treated ankle fracture and the age- and gender matched norms of the Swedish population

**SF-36 sub-scales**	**6-month follow-up **(n = 31) Mean (SD)	**12-month follow-up **(n = 31) Mean (SD)	**Norms of the Swedish population **Mean (95% CI)	p-values* 6-month follow-up	**p-values¤ 12-month follow-up**
**Physical functioning**	71.6 (24.2)	77.5 (20.6)	73.8 (71.6–76.0)	0.717	0.514
**Role physical**	56.3 (44.3)	67.9 (39.7)	64.9 (61.4–68.5)	0.446	0.785
**Bodily pain**	67.9 (20.0)	65.6 (22.9)	70.6 (68.3–72.9)	0.602	0.427
**General health**	76.7 (20.7)	76.8 (16.7)	66.8 (64.8–68.8)	0.075	**0.044**
**Vitality**	71.5 (18.2)	72.1 (18.7)	68.7 (66.5–70.9)	0.554	0.503
**Social functioning**	84.4 (23.9)	90.2 (15.6)	87.4 (85.5–89.3)	0.621	0.518
**Role emotional**	68.8 (43.0)	76.9 (34.4)	77.2 (74.1–80.3)	0.444	0.977
**Mental health**	82.8 (15.8)	77.7 (18.2)	83.5 (81.8–85.1)	0.852	0.256

* p-values comparing patients results to normative data at 6-month follow-up;

¤ p-values comparing patients results to normative data at 12-month follow-up

### Supplementary questions

Out of the 50 patients 30 (60%) experienced functional ankle instability, mostly when walking on uneven ground, at 6-month follow-up and 24 (52%) had the experience at 12-month. Before injury 41 (82%) were physically active, 24 at a lower level and 16 at a moderate and one at high level. After 6 months 13/41 had returned to the same activity level and 18/41 after 12 months. Ten subjects used a walking aid before injury, mostly a walking frame. After 6-months 13 were in the need of walking aid and one needed a wheel-chair.

### Radiological examination

Thirty-nine (78%) of the fractures were SE IV, showing the same injury pattern; dorsally or laterally dislocated talus and a malleolar fragment. The postoperative radiographic results showed complete ankle joint congruency in 40 (80%) ankles, < 2 mm in-congruency in 6 (12%) and > 2 mm in-congruency in two and in two cases the information was missing. Fracture reduction was complete in 23 (46%) ankles, < 2 mm displacement in 14 (28%), > 2 mm in 13 (26%) with no differences between men and women (p = 0.531). A new radiographic examination was performed in 42 subjects 16 (range 12–32) (IQR 8.3) months after surgery and at that time all fractures were healed and 37/42 ankles showed joint congruency. Fracture reduction was complete in 31/42 ankles, < 2 mm displacement in 4 and > 2 mm in 7. Four patients had discrete signs of osteoarthritis (a loss of joint space less than 50%), two had moderate osteoarthritis (a loss of joint space of more than 50% but no bone-to-bone contact) and one had severe osteoarthritis (bone-to-bone contact) with no differences between men and women (p = 0.603).

## Discussion

The results of this study indicate that a majority of patients continue to have symptoms and reported functional limitations still one year after surgically treated ankle fractures. Sixty percent of the patients or more reported ankle pain, swelling and problems when using stairs and reduced activities of daily life still one year after injury. Eighty-two percent were physically active before injury but less than half of the patients had returned to their pre-injury activity level one year after. Health-related quality of life was influenced in women but not in men. Most of the fractures were bi-or trimalleolar and less than half of the fractures were reduced completely postoperatively.

Subjective ankle function as measured by OMAS, LAS and self-rated ankle function increased between the two follow-ups in the total sample. In women the results from OMAS and LAS increased significantly but in men no differences were detected. However, due to the low number of men there could be a risk analysing each gender. It cannot be excluded that differences would have been found also in men in a larger sample (type II error). The distribution between men and women in the studied group could be expected as ankle fractures occur in men more often in younger ages, whereas women injure more frequently after the age of 50 [28].

Many studies have evaluated outcome and subjectively scored function after surgically treated ankle fractures [3-7,9,10,20,29-31], but none of these studies have evaluated outcome in the elderly specifically. The only study found publishing OMAS results for different age groups is that of Lash *et al.* including 74 patients, 22 men and 52 women, 22–89 years of age. A mean score of 76 points was found in the age group 17–40, 79 points in the age group 41–60 and 84 points in the age group 61–89 two years after injury showing improved results with age [7]. However, the differences were not significant and in that study only two thirds of the fractures were surgically treated [7].

Earlier studies including also younger age groups showed better function as measured by OMAS one year after injury compared to the results in the present study. Hedström *et al.* evaluated lateral malleolar fractures and found an OMAS of median 88 points in patients with a mean age of 42 years. Although the ages in that study ranged from 16–71 years the results were presented as median values for the total sample [3]. Lehtonen *et al.* studied surgically treated ankle fractures in patients mean 41 years of age and found a median OMAS of 90p [31] and van Laarhoven *et al.* found an OMAS of median 95 points in patients with a mean age of 36 (range 17–77) years showing excellent scored function. Not either in that study the results were presented for different age groups [29]. Tropp *et al.* found a mean value of 91 points in a group of 30 subjects with uni-, bi- and trimalleolar ankle fractures with a mean age of 26 [4]. Both Nilsson *et al.* and Egol *et al.* reported that an age over 40 increased the risk of poorer scored function one year after surgery [9,10] and also three years after [9]. The OMAS results from the group over 40 years of age were comparable to the group in the present study. These outcomes support the findings that younger age groups reach better function and that adult persons with the same type of fractures should not be regarded as a homogenous group. Age should be taken into consideration when evaluating results after ankle fracture.

The use of OMAS in the elderly could be questioned as it includes the items 'jumping', 'running' and 'squatting', functions that might be difficult only due to higher age. Because of that not only the median values of OMAS but also the results from the separate items have been analysed and presented as it gives more detailed information about function. The experience of pain, stiffness, problems when stair-walking and reduced activity of daily life were more frequent in the present age group compared to the results including patients 18–64 years of age [9]. However, the impossibility to jump, run and squat were most frequent reported and it can not be excluded that higher age was a contributing reason for this.

There was a significant correlation between OMAS and LAS but the figures from LAS were higher and we believe that one reason might be the three items mentioned above. LAS include some sort of expected recovery as well and it is possible that subjects at higher ages do not expect to reach full recovery after a surgically treated ankle fracture. This might explain the diversity between the results from the two scores. It is possible that the patients had adapted to and accepted a lower activity level.

Egol *et al.* investigated quality of life in surgically treated ankle fractures already at baseline using Short Musculoskeletal Function Assessment (SMFA) and concluded that ankle fracture patients are in general a healthy group compared to normative data [10]. The results from our study confirm this assertion as eighty percent had been physically active before injury, which might be a higher percentage than normative in the studied age group. Furthermore in men no impairments regarding health-related quality of life was found, in contrary the dimension 'general health' was even higher than the norms at 12-month. In women however, there were differences in SF-36 below normative data regarding 'physical function' and 'role physical' and 'role emotional' at 6-month follow-up. As most questions in the physical subscales concern activities loading the lower extremities it could be expected being impaired as soon as six months after injury. There were also differences in women also in the 'role emotional' subscale. Physical limitations in persons who normally are physically active may influence their emotional life. However, no limitations were seen in the rest of the mental health subscales. Although most subjects had been physically active before injury less than half of them had been able to return to their pre-injury physical activity level still one year after the fracture. We found similar results in one of our earlier studies including patients 18–64 years of age [32]. It is probably more important for elderly to recover function as fast as possible since bone mass density, proprioception and muscle force already are decreased due to the higher age. Inactivity might lead to decreased physical function which in turn might be a risk of new falls [33].

Until recently ankle fractures have not been regarded as fragility fractures [34-36] but a review by Court-Brown and Caesar (2006) revealed that at least bi-and trimalleolar fractures should be regarded as osteoporotic as they occur more often in older women and follow the pattern of fragility fractures [37]. Many authors have also presented that severely dislocated fractures are coincided with poor outcome [7,13,38]. The large numbers of severely dislocated fractures in the studied group, many not possible to reduce completely, might be one explanation to the poor outcome. Furthermore almost 80 percent reported different kinds of co-morbidities and half of them were impairments in the musculoskeletal system. It is likely to believe that these figures are representative for the studied age group and this could be another explanation to the poor outcome.

Limitation of this study is the lack of a non-injured control group and information of baseline function before injury. However, the age- and gender matched normative data of the Swedish population regarding SF-36 was available. Furthermore we recorded some pre-injury information about physical activity level although first at six month follow-up and we are aware that this information might be uncertain. Finally our study included a follow-up time of only one year. It is probably even more important to follow the elderly over a longer time as functions recover more slowly compared to younger age groups. Future studies are needed to elucidate whether outcomes improve or impair with time.

## Conclusion

In conclusion, one year after surgically treated ankle fractures a majority of patients continue to have symptoms and reported functional limitations. However, SF-36 scores indicate that only females had a functional status lower than age- and gender matched normative data. Sixty percent or more of the patients reported pain, swelling, problems when stair-climbing and had reduced activities of daily life. Less than half of the subjects had returned to their pre-injury physical activity level. Most fractures were bi- and trimalleolar and most cases having a postoperative residual displacement. Future studies in the elderly are needed including larger samples able to analyse if subgroups are at higher risk of poor outcome. Furthermore the results should be followed over a longer time to elucidate whether outcomes improve or impair with time.

## Competing interests

The author(s) declare that they have no competing interests.

## Authors' contributions

GN participated in the design of the study, collected the data, performed the statistical analyses, and drafted the manuscript. KJ examined all the radiographs. CE participated in the design of the study, and in the progress and revision of the manuscript. ME was responsible for identifying and including the patients, and participated in the progress and revision of the manuscript. All authors read and approved the final manuscript.

## Pre-publication history

The pre-publication history for this paper can be accessed here:


